# Modeling of Surface Topography after Milling with a Lens-Shaped End-Mill, Considering Runout

**DOI:** 10.3390/ma15031188

**Published:** 2022-02-04

**Authors:** Karol Żurawski, Piotr Żurek, Andrzej Kawalec, Anna Bazan, Adam Olko

**Affiliations:** 1 Department of Manufacturing Techniques and Automation, Faculty of Mechanical Engineering and Aeronautics, Rzeszów University of Technology, ul. Wincentego Pola 2, 35-959 Rzeszów, Poland; p_zurek@prz.edu.pl (P.Ż.); ak@prz.edu.pl (A.K.); abazan@prz.edu.pl (A.B.); 2 Pratt & Whitney Rzeszów S.A., ul. Hetmańska 120, 35-078 Rzeszów, Poland; adam.olko@prattwhitney.com

**Keywords:** surface topography, five-axis machining, lens-shape end-mill, milling process parameters, response surface model

## Abstract

The paper presents a method of forecasting the product surface topography after five-axis machining with a lens-shaped end-mill. Surface roughness is one of the key parameters considered when assessing the effectiveness of the machining process, especially in the aviation, automotive, tooling and medical equipment industries. The developed method, the first published, presented in the paper is based on the analytical equations of the trajectory of the cutting edge motion, on the basis of which the cutter action surface is generated. The developed model takes into account: cutting depth, cutting width, feed, lead angle and radial runout. Experimental studies were conducted using three different materials: 40HM steel, Al7035 aluminum alloy and Ti Grade 5 titanium alloy. Various values of the cutting width parameters and different feeds were used in the tests. Based on the results of the experimental tests, an empirical model (response surface model) was determined and was then used to verify the simulation model. The simulation results and the results of experimental tests were compared and conclusions were drawn regarding the developed models. The developed models supported by numerical simulation can be used to approximately estimate the influence of the width of cut $b_{r}$ and feed $f_{t}$ on selected height characteristics $S_{a}$ and $\hat{Sz}$ of the geometric structure of the surface (GSS) after machining with a lens-shaped end-mill in terms of the process parameters adopted in the tests. It was found that the influence of the $f_{t}$ on the $S_{a}$ and $\hat{Sz}$ is greater for small values of $b_{r}$. The effect of $b_{r}$ is greater with lower $f_{t}$ values. The cutting width $b_{r}$ has the greatest influence on $S_{a}$ and $\hat{Sz}$, and $f_{t}$ and the interaction of these parameters has the least influence.

## 1. Introduction

The five-axis milling technology is used in the production of parts with very high design requirements [1,2,3]. These requirements include surface roughness, which is of key importance for the durability and reliability of manufactured parts [4,5]. It affects the coefficient of friction [6,7,8], fatigue strength [9,10], wear resistance [11,12,13], corrosion [14,15] and creep resistance [16,17]. These properties are extremely important for components operating in very difficult conditions, i.e., working under heavy loads, at high temperatures and pressures, and in an aggressive environment.

**Figure 1:**
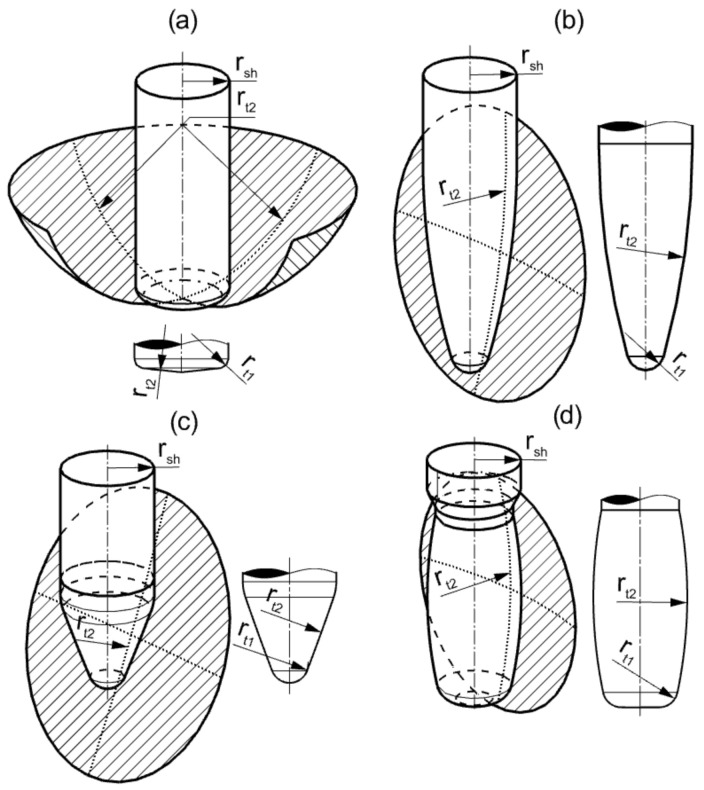
Circle segment end mills: (**a**) lens shape, (**b**) oval form, (**c**) taper form, and (**d**) barrel shape.

Five-axis milling is very often used in the production of components with complex shapes, where the manufacturing cost is many times greater than the value of the blank. It belongs to the group of technologies called high-value-added manufacturing. Therefore, new solutions are still being sought to increase the efficiency of the milling process [3,18,19]. Due to the dynamic development of various Computer-Aided Manufacturing (CAM) applications, increasingly often specialized tools with non-standard contours are used in machining, which replace cylindrical and spherical mills in selected specific machining tasks. One of such solutions are circular-shape end-mills [20,21,22,23,24,25,26,27,28,29,30]. The outline of these tools is defined by an arc with a radius $r_{t2}$ much larger than the tool shank radius $r_{sh}$. Depending on the position of the arc $r_{t2}$ on the cutter contour, different cutter geometries can be obtained (Figure 1).

**Figure 2:**
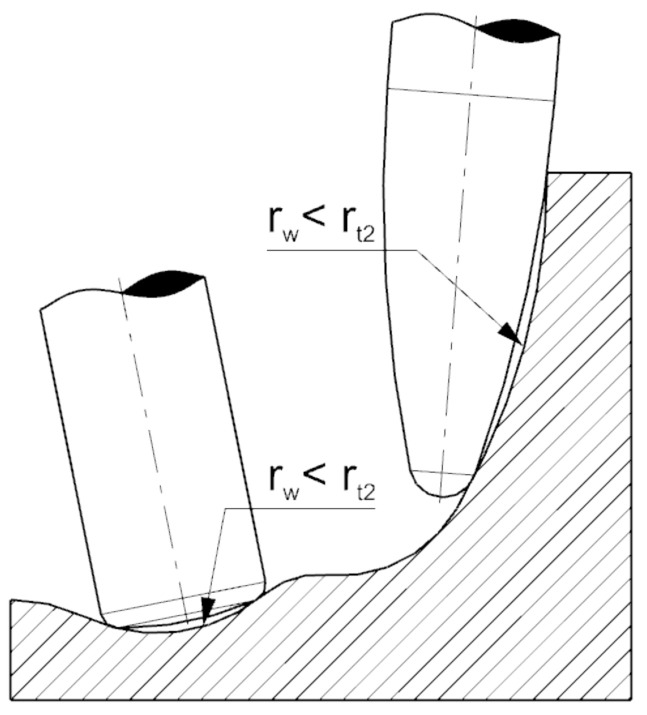
Example of circle segment end mills limitations.

The use of circular cutters allows a significant reduction in the number of machining passes while obtaining the required surface roughness after machining. The main disadvantage of the above-mentioned tools is their limited applicability due to the shape of the workpiece (Figure 2).

The importance of the issue of surface topography modeling is reflected in a large number of scientific studies. Kim et al. presented a method, based on the solution of Koret et al., of modeling the surface topography after three-axis milling with cylindrical tools, taking into account the radial runout [31,32].

Jung et al. proposed in their works a method of forecasting surface roughness after machining with a spherical cutter, which consists in analytically determining the so-called ridge curves. The model developed using this method includes, inter alia, influence of feed and width of machining passes. The developed method was compared with other methods of surface topography determination and was also verified in experimental studies, during which the Al2024 aluminum alloy was processed [33,34].

Nespor et al. presented a model of the Ti6Al4V alloy surface topography after re-contouring with spherical cutters. In the developed model, the rotational movement of the tool, feed, deflection of the tool, radial runout and irregularity of the cutting edge were taken into account [35].

Hao et al. proposed a method for determining the surface roughness after a spherical milling cutter of thin-line elements. In their model, they took into account the trajectory of the tool, the deformation of the machined surface as a result of the cutting force components and tool wear. The model was verified in experimental tests in which grade 45 steel was processed with the use of different values of cutting speed $v_{c}$, depth of cut $a_{p}$ and feed $f_{n}$ [36].

In the work of Ehsan et al. there is presented a model of surface topography for spherical cutters in five-axis machining. The proposed model is based on determining the points of intersection of the trajectory of the cutting edge motion through a series of planes perpendicular to the feed vector. Thanks to this, it is possible to determine the mean value Sa and mean square value Sq taking into account the rotational movement of the tool, feed, number of teeth, depth and width of cut, radial runout. The influence of the lead angle and the inclination of the tool was also taken into account. The simulation results were compared with the experimental results obtained while machining the 7050 aluminum alloy at different feeds and values of the lead angle and inclination [37].

Gdula developed in his work an empirical model of the roughness of free surfaces after five-axis machining with a toroidal cutter. This model takes into account the influence of the value of the surface curvature radius and the toroidal milling angle. It was developed on the basis of the results obtained during the treatment of Inconel 718 alloy [38].

Urbikain et al. presented in their work a model of surface roughness for cutters with oval-forms in five-axis machining. The developed model is based on determining the trajectory of the cutting edge movement, and then determining which part is contained in the volume of the blank. This model takes into account the influence of feed, depth of cut, lead and shear angles, radial runout and tool helix angle. The analysis takes into account the case of a single machining step. The created model was verified in experimental studies in which the Al7075T aluminum alloy was processed using two tool diameters, different feeds and angles of guidance and inclination [39].

In another work by Urbikain et al. there is presented a geometric and empirical model of the surface roughness after barrel-shape machining. In the case of the geometric model, the method developed in [39] was used. In this case, the feed, radial runout and tool helix angle were also taken into account. The empirical models were made dependent on the values of the cutting speed, feed and width of cut. Both the geometric and empirical model has been verified in experimental studies in which different values of cutting speed, feed and cutting width were used. The materials treated were Al7075 T6 aluminum alloy and Ti6Al4V titanium alloy. In both the developed models and the experimental tests there was analyzed the case of a single machining step [40].

Among the tools with a circular outline, the lens-shaped end-mill can be distinguished. Despite the available solutions of this type of cutters from tool manufacturers, there is no published research on this subject available. The shape of the face of the lens-shaped end-mill makes it a potentially attractive tool for machining curvilinear surfaces with a radius of curvature greater than the radius $r_{t2}$ due to the significantly reduced number of machining passes in relation to spherical cutters with the assumed roughness of the machined surface. The aim of this work was to develop a simulation model of the surface texture obtained as a result of five-axis machining with a lens-shaped end-mill as well as its experimental verification.

## 2. Materials and Methods

### 2.1. Modeling Assumptions

The model assumptions can be formulated as the following conditions:

- The shape of the cutter is described with the dimensions given in Figure 1.
- The cutting edge helix angle ζ is specified.
- The tool moves taking into account the lead angle α.
- The tool and workpieces are rigid.
- Radial runout is defined as the increment $\Delta r_{te}$ of the effective tool radius $r_{te}$.
- Variable parameters are feed $f_{t}$ and width of cut $b_{r}$. The remaining parameters in the equations are constant.

### 2.2. Topography Simulation Model

The trajectory of any point $P\left(t\right)={\left[x_{0}\left(t\right),y_{0}\left(t\right),z_{0}\left(t\right)\right]}^{T}$ located on the cutting edge of a milling cutter performing only rotary motion can be described by the following system of equations:

(1) $$\left\{\begin{array}{cc} x_{0}\left(t\right)=r_{te}\cdot \cos\left(\kappa \cdot t+\theta -\psi \right) & \\ y_{0}\left(t\right)=-r_{te}\cdot \sin\left(\kappa \cdot t+\theta -\psi \right) & \\ z_{0}\left(t\right)=h-r_{t2} & \end{array}\right.$$

where

$x_{0}\left(t\right)$, $y_{0}\left(t\right)$, $z_{0}\left(t\right)$—coordinates of any point P(t) located on the cutting edge of a milling cutter,

*h*—the distance of a point on the cutting edge from the end point on the tool axis along that axis,

$r_{t2}$—the outline radius of the cutting edge,

$r_{te}$—the effective radius of the tool,

t∈<0,1>—parameter specifying the position of a point on the curve,

θ—the angle between the cutting edges.

**Figure 3:**
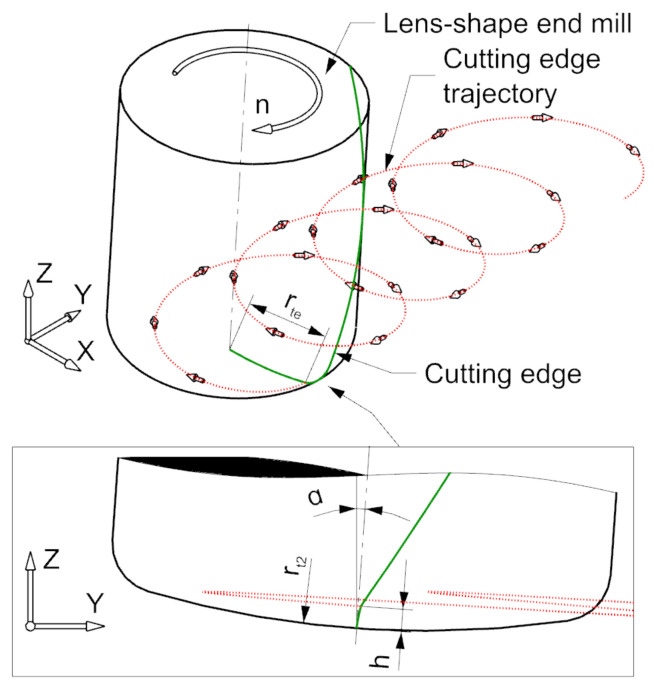
The trajectory of the point located on the edge of the lens-shaped end-mill.

In the case of machining with a lens milling cutter, the effective radius $r_{te}$ (Figure 3) of the tool face defined by radius $r_{t2}$ can be described by the formula [41]:

(2) $$r_{te}=\sqrt{r_{t2}^{2}-{\left(r_{t2}-h\right)}^{2}}$$

Additionally, the total rotation angle κ of the tool can be determined by the equation:

(3) $$\kappa =2\cdot \pi \cdot i_{t}$$

where $i_{t}$ is the number of rotations of the tool.

In addition, the initial angular shift ψ resulting from the cutting edge inclination angle ζ should be taken into account, the value of which can be described by the formula:

(4) $$\psi =\frac{h\cdot \tan(\zeta )}{r_{t2}}\cdot \frac{180}{\pi }$$

Introducing some disturbance in the XY plane in the form of, e.g., radial runout $\Delta r_{te}$ transforms Equations (1) and (4) to the following form:

(5) $$\left\{\begin{array}{cc} x_{0}\left(t\right)=\left(r_{te}+\Delta r_{te}\right)\cdot \cos\left(\kappa \cdot t+\theta -\psi \right) & \\ y_{0}\left(t\right)=-\left(r_{te}+\Delta r_{te}\right)\cdot \sin\left(\kappa \cdot t+\theta -\psi \right) & \\ z_{0}\left(t\right)=h-r_{t2} & \end{array}\right.$$

(6) $$\psi =\frac{h\cdot \tan(\zeta )}{r_{t2}+\Delta r_{te}}\cdot \frac{180}{\pi }$$

The lead angle α is defined as the rotation of the tool axis in the plane formed by the normal vector of the machined surface and the feed vector. If we assume that the normal direction is parallel to the *Z* axis and the tool feed will be in the direction of the *Y* axis, then taking into account the lead angle α in Equation (5) can be achieved by rotating around the *X* axis [39]:

(7) $$\left[\begin{array}{c} x(t)\\ y(t)\\ z(t) \end{array}\right]=\left[\begin{array}{ccc} 1 & 0 & 0\\ 0 & \cos(\alpha ) & \sin(\alpha )\\ 0 & -\sin(\alpha ) & \cos(\alpha ) \end{array}\right]\left[\begin{array}{c} x_{0}\left(t\right)\\ y_{0}\left(t\right)\\ z_{0}\left(t\right) \end{array}\right]$$

where x(t), y(t), z(t) are the coordinates of any point located on the cutting edge of a milling cutter after consideration of the lead angle α.

As a result, the trajectory equation takes the following form:

(8) $$\left\{\begin{array}{cc} x\left(t\right)=\left(r_{te}+\Delta r_{te}\right)\cdot \cos\left(\kappa \cdot t+\theta -\psi \right) & \\ y\left(t\right)=-\left(r_{te}+\Delta r_{te}\right)\cdot \sin\left(\kappa \cdot t+\theta -\psi \right)\cdot \cos\left(\alpha \right)+\left(h-r_{t2}\right)\cdot \sin\left(\alpha \right) & \\ z\left(t\right)=\left(r_{te}+\Delta r_{te}\right)\cdot \sin\left(\kappa \cdot t+\theta -\psi \right)\cdot \sin\left(\alpha \right)+\left(h-r_{t2}\right)\cdot \cos\left(\alpha \right) & \end{array}\right.$$

We assume that the origin of the coordinate system is the cutter’s initial point of contact with the workpiece. Therefore, in Equation (8), the shift in the *Z* axis by the value of the radius $r_{t2}$ should be taken into account. As a result we get

(9) $$\left\{\begin{array}{cc} x\left(t\right)=\left(r_{te}+\Delta r_{te}\right)\cdot \cos\left(\kappa \cdot t+\theta -\psi \right) & \\ y\left(t\right)=-\left(r_{te}+\Delta r_{te}\right)\cdot \sin\left(\kappa \cdot t+\theta -\psi \right)\cdot \cos\left(\alpha \right)+\left(h-r_{t2}\right)\cdot \sin\left(\alpha \right) & \\ z\left(t\right)=\left(r_{te}+\Delta r_{te}\right)\cdot \sin\left(\kappa \cdot t+\theta -\psi \right)\cdot \sin\left(\alpha \right)+\left(h-r_{t2}\right)\cdot \cos\left(\alpha \right)+r_{t2} & \end{array}\right.$$

Introducing into Equation (9) the displacement in the direction of the *Y* axis resulting from the tool feed per revolution $f_{n}$, we obtain the final form of the trajectory equations of any point of the cutting edge of the front part of the lens milling cutter taking into account the radial runout (Figure 3):

(10) $$\left\{\begin{array}{cc} x\left(t\right)=\left(r_{te}+\Delta r_{te}\right)\cdot \cos\left(\kappa \cdot t+\theta -\psi \right) & \\ y\left(t\right)=-\left(r_{te}+\Delta r_{te}\right)\cdot \sin\left(\kappa \cdot t+\theta -\psi \right)\cdot \cos\left(\alpha \right)+\left(h-r_{t2}\right)\cdot \sin\left(\alpha \right)+f_{n}\cdot i_{t}\cdot t & \\ z\left(t\right)=\left(r_{te}+\Delta r_{te}\right)\cdot \sin\left(\kappa \cdot t+\theta -\psi \right)\cdot \sin\left(\alpha \right)+\left(h-r_{t2}\right)\cdot \cos\left(\alpha \right)+r_{t2} & \end{array}\right.$$

**Figure 4:**
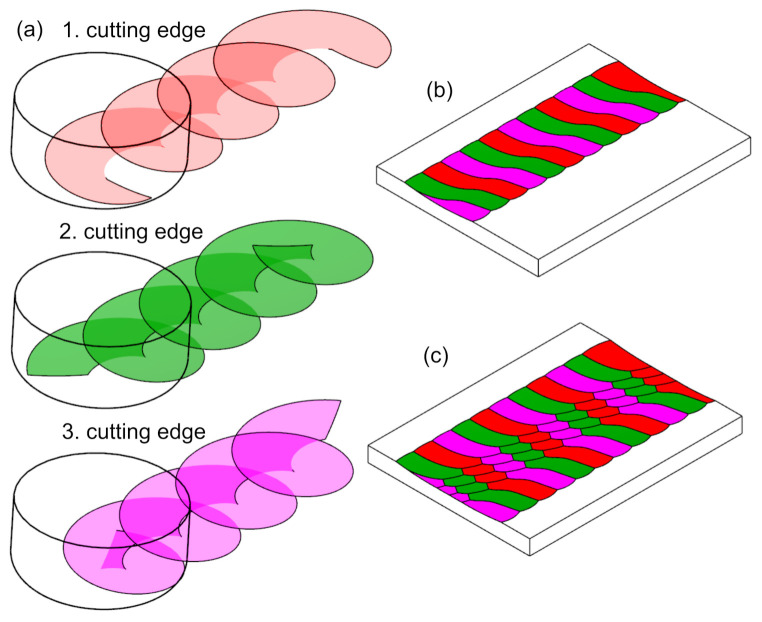
(**a**) Model of the surface of action of individual cutting blades. Surface texture model: (**b**) “one path” (**c**) “several paths”.

**Figure 5:**
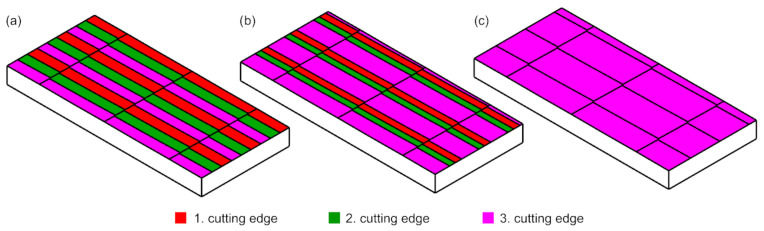
Surface texture model for $f_{t}$ = 0.06 mm, $b_{r}$ = 0.4 mm and the radial runout of the tool $\Delta r_{te}$ (**a**) $\Delta r_{te}=0$ mm (**b**) $\Delta r_{te}=0.001$ mm (**c**) $\Delta r_{te}=0.01$ mm.

Using Equation (10) in the workspace of the Siemens NX system (Siemens, Munich, Germany), a geometry was created reflecting the surfaces of action of individual cutting blades. By using them to trim the plane, a model of the surface topography was obtained (Figure 4 and Figure 5).

### 2.3. Experimental Research

**Figure 6:**
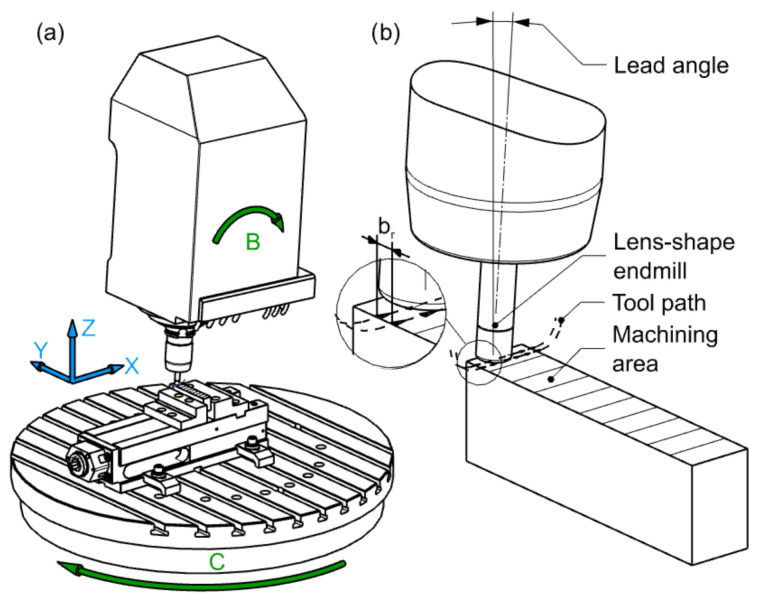
Scheme of (**a**) machine tool configuration with degrees of freedom along the X, Y and Z axes as well as rotational degrees of freedom B and C, carried out by the table or the head of the machine tool and (**b**) lens-shaped end mill machining.

**Figure 7:**
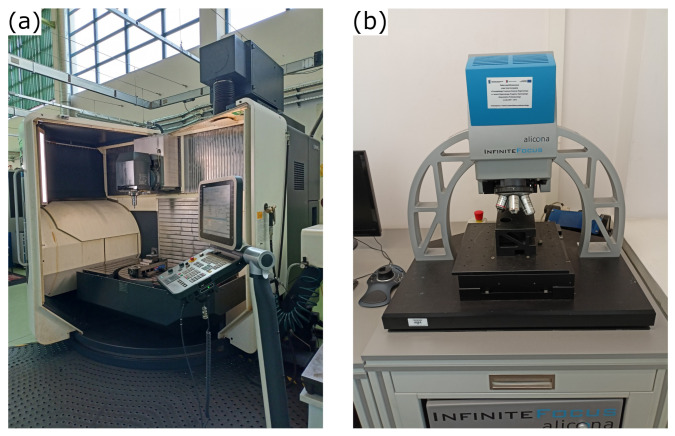
Research–measurement stand: (**a**) DMU 100 monoBLOCK milling center, (**b**) 3Dsystem InfiniteFocus Alicona optical microscope.

The experimental investigations were carried out on the DMG monoBlock 100 five-axis machining center (DMG, Pleszew, Poland) (Figure 6 and Figure 7). A lens-shaped end-mill EMUGE FRANKEN 3544L.10020A (EMUGE Werk Richard Glimpel, Lauf, Germany) with radius $r_{t2}=20$ mm was used for processing. Three types of materials representing various ISO material grades were used for the tests [42]:

- 40HM steel (Stalton, Rzeszów, Poland)—grade ISO P. Metals of this grade are characterized by good machinability. They are, however, quite diverse due to the different carbon content in the alloy. These are the materials most commonly used in industry. Depending on the alloying elements, for example the following products are made of these materials: shafts, crankshafts, gears, discs, rotors.
- Al7035 aluminum alloy (EINSAL East, Mikołów, Poland)—grade ISO N. These alloys are easy to machine and allow for high-performance machining. They are used to make engine blocks, bodies, structural elements of aircraft fuselages. In addition to aluminum, this class includes materials such as brass and copper.
- Ti Gr 5 titanium alloy (WOLFTEN, Wrocław, Poland)—grade ISO S. It is characterized by difficult machinability. They have poor thermal conductivity and therefore have a high processing temperature. They are used to make elements such as blades of aircraft engines, rotors, parts of the landing gear or medical implants. Other heat-resistant alloys, such as nickel or cobalt, also fall into this grade.

Among three considered materials steel 40HM was in softened state. The remaining two materials Al7075 T6 aluminum alloy and Ti Gr 5 titanium alloy were not heat treated. Each of the materials was in the initial state without any thermo-chemical preliminary treatment. A flood cooling method was used in machining process.

The research sample was a cuboid with separated 10 planes. Each of these areas was processed with different feed parameters $f_{t}$, cutting width $b_{r}$. The cutting speed was selected for each material according to the recommendations of the tool manufacturer (Table 1). It is important that the working motion started 20 mm before the beginning of the test field, which guaranteed the achievement of the assumed feed rate (Figure 6).

Then, surface topography measurements were performed on all the samples. They were realized on an optical microscope 3Dsystem G4 InfiniteFocus Alicona (Alicona Imaging, Raaba, Austria) with the “focus variation” method (Figure 7). An ×50 lens (Alicona Imaging, Raaba, Austria) was used, vertical resolution was 50 nm, horizontal resolution was 2.135 μm, pixel size was 0.35 μm × 0.35 μm. The measurement area was 0.22 mm × 3.3 mm.

### 2.4. Design of Experiment and Statistical Analysis

Design of experiment and statistical analysis of data was done using the JMP 12 software package (SAS Institute, Cary, NC, USA). The significance level $\alpha_{sl}=0.05$ was assumed for all tests. The input variables were material of the workpiece, feed rate $f_{t}$ and cutting width $b_{r}$ (Table 2). The experiment plan consisted of 3 separate central compositional plans for each material (10 trials each), in which the input variables were $f_{t}$ i $b_{r}$. The overall experiment plan consisted of 30 research trials.

It was assumed that the output variables will be two surface texture height parameters. The first parameter of averaging nature Sa —the arithmetical mean height of the scale-limited surface, the second one takes into account the maximum deviations from the mean area Sz —the maximum height of the scale-limited surface [43]. Due to the optical method of measuring the surface topography, the analysis of the Sz parameter would not be advisable. Optical measurements, including the focus variation method, are prone to the presence of artifacts in the form of unusual peaks, which greatly distort the actual Sz values of the analyzed surface. In order to avoid possible errors when reducing areas with artifacts from topography maps, instead of the Sz parameter, the sum of parameters related to the bearing area curve (BAC; the Abbott–Firestone curve) was analyzed $\hat{\mathrm{Sz}}=\mathrm{Spk}+\mathrm{Sk}+\mathrm{Svk}$ (reduced maximum height) [44]. Parameters such as reduced peak height Spk and reduced dale height Svk largely retain information about the height of peaks and the depth of pits, but also filter information about unusual deviations from the mean area. Parameter Sk represents core roughness depth computed on the basis of the BAC.

Based on empirical data, for a given surface texture parameter, a response surface model was developed for three input variables of the general form [45,46]:

(11) $$y=\beta_{0}+\sum_{i=1}^{3}\beta_{i}x_{i}+\sum_{i=1}^{3}\sum_{j=1}^{3}\beta_{ij}x_{ij}+\sum_{i=1}^{3}\beta_{ii}x_{i}^{2}$$

Then, statistically insignificant factors were removed from the models by means of back regression. For the developed models, the presence of influencing observations was tested on the basis of Cook’s distance, assuming a limit value D=0.7. The statistical significance of the model was tested using the analysis of variance. The normality of the residual distribution was tested by the Shapiro-Wilk test, and their mean value (which should be 0) by the Student’s *t*-test.

The normality of the distributions was tested with the Shapiro–Wilk test. This test is based on the study of the correlation between empirical data and corresponding normal scores. If this correlation is large, it is assumed that the test sample comes from a normally distributed population [47].

The models were verified with k-fold cross-validation with stepwise regression, assuming k=5. In the process of cross-validation, the samples were randomly divided into 5 parts. Then, by means of back regression, the models were derived five times on the basis of 4 parts of the samples, and the remaining part of the samples (different each time) were used to verify the model [48,49].

## 3. Results

**Figure 8:**
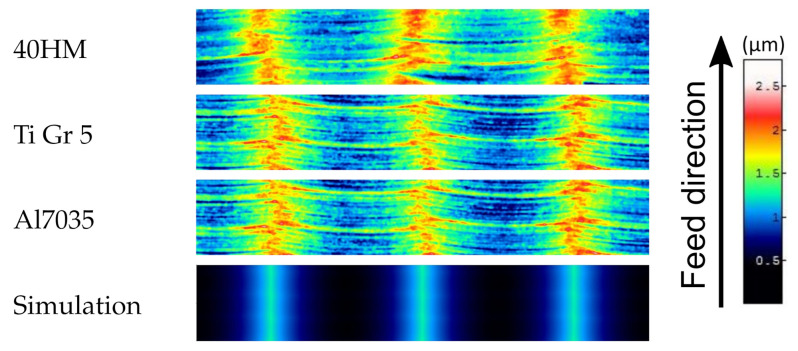
Surface parts with dimensions of 0.2 mm × 1.2 mm from the measured surface topographies of samples made of various materials and the surface obtained from simulation with milling parameters $b_{r}$ = 0.4 mm, $f_{t}$ = 0.06 mm.

**Figure 9:**
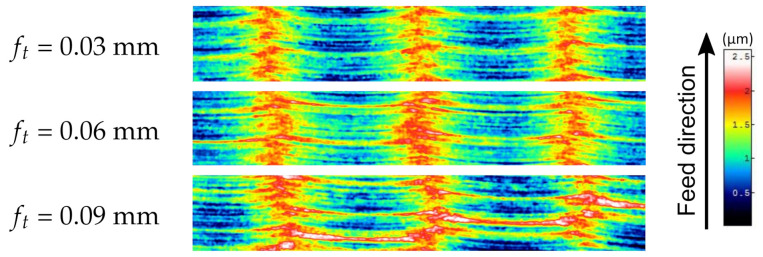
Surface parts with dimensions of 0.2 mm × 1.2 mm from the measured surface topographies of samples made of titanium alloy Ti Gr 5 after milling with a cutting width of $b_{r}$ = 0.4 mm and different values of feed $f_{t}$.

**Figure 10:**
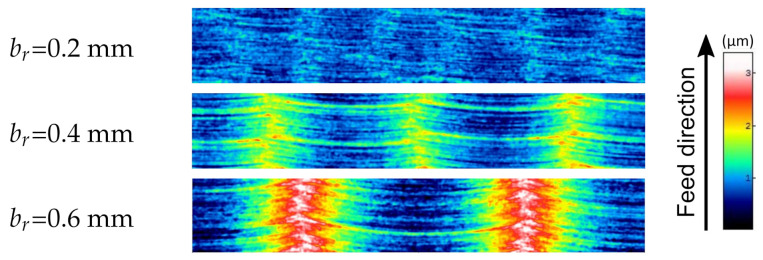
Surface parts with dimensions of 0.2 mm × 1.2 mm from the measured surface topographies of samples made of titanium alloy Ti Gr 5 after milling with feed $f_{t}$ = 0.06 mm and different values of cutting width $b_{r}$.

The Figure 8, Figure 9 and Figure 10 show fragments of selected measured surfaces and one example of a surface after simulation. The surfaces obtained as a result of empirical and simulation tests are characterized by a distinct periodic structure. The size of the period depends on the milling width $b_{r}$ (Figure 10). The observation of the topography maps also confirms that the $b_{r}$ parameter has a very significant influence on the height parameters of the surface topography. The surfaces of samples made of various materials (Figure 8) after milling are characterized by a similar microgeometry. On the simulated surface, there are irregularities resulting from the successive passes of the tool. However, there are no irregularities in the direction of the feed rate.

In the case of both developed models, i.e., for the dependent variable Sa and $\hat{\mathrm{Sz}}$, the significant factors turned out to be the feed rate and the width of the cut and their interaction (Table 3 and Table 4). The type of processed material and its interactions with the tested parameters, i.e., feed $f_{t}$ and cutting width $b_{r}$, of the milling process turned out to be statistically insignificant. No influential observations were found for either model. The probability values determined in the analysis of variance for each of the models were lower than $2\times 10^{-17}$. The residuals indicated a normal distribution with an average of 0.

In order to assess the quality of the model fit to the data, the coefficient of determination for the final models developed on the basis of 30 trials was determined. This coefficient, called fit coefficient $R^{2}$, informs about the quality of fitting the models to empirical data. It is determined on the basis of the coefficient of determination $R^{2}$, taking into account the number of trials and the number of equation parameters to be calculated [45]:

(12) $$R^{2}=\frac{SS_{R}}{SS_{T}}=1-\frac{SS_{res}}{SS_{T}}$$

where

$SS_{R}$—explained regression sum of squares

$SS_{T}$—total sum od squares

$SS_{res}$—residual sum of squares

In order to find out how well the model responds to data that it has not seen—that is, the predictability of the model the adjusted fit coefficient was calculated for 5 models created during cross-validation (each of these 5 models was developed on the basis of 24 trials). This coefficient, called adjusted fit coefficient and denoted $R_{adj}^{2}$ was computed according to the formula below [45]:

(13) $$R_{adj}^{2}=1-\frac{SS_{res}/\left(n-p\right)}{SS_{T}/\left(n-1\right)}=1-\left(\frac{n-1}{n-p}\right)\left(1-R^{2}\right)$$

where

*n*—sample size

*p*—total number of coefficients number of explanatory variables plus constant term

The adjusted fit coefficient $R_{adj}^{2}$ for the Sa focused model was 0.97, and for the $\hat{\mathrm{Sz}}$ model was 0.96. The models could therefore be considered to be very well suited to the empirical data.

**Figure 11:**
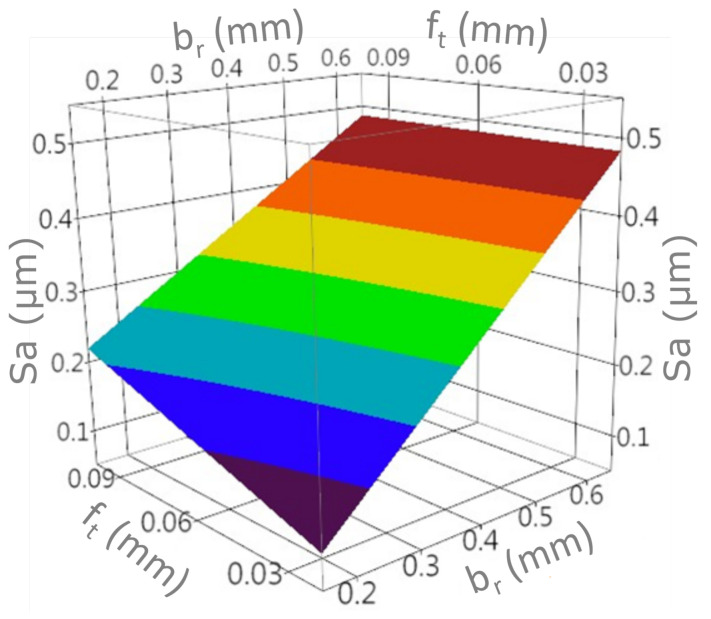
The response surface for the model with the Sa output variable.

**Figure 12:**
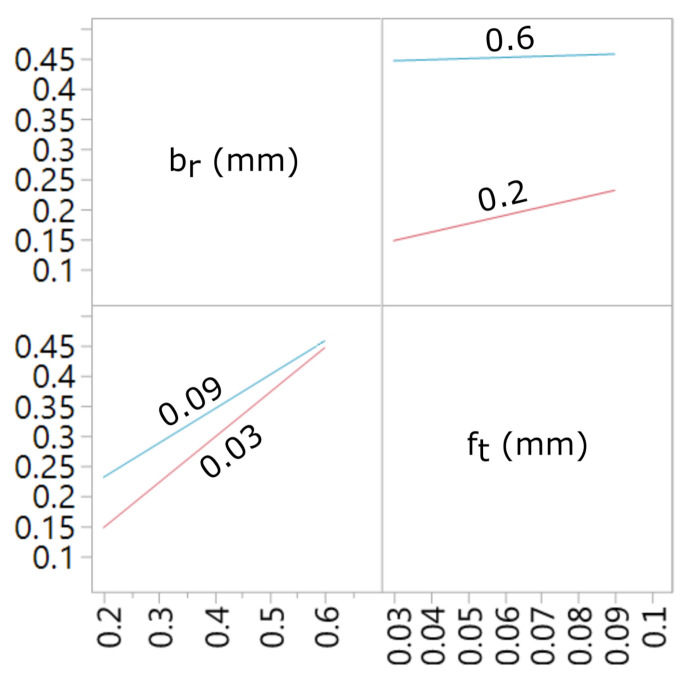
Interaction plot for a model with the Sa output field.

**Figure 13:**
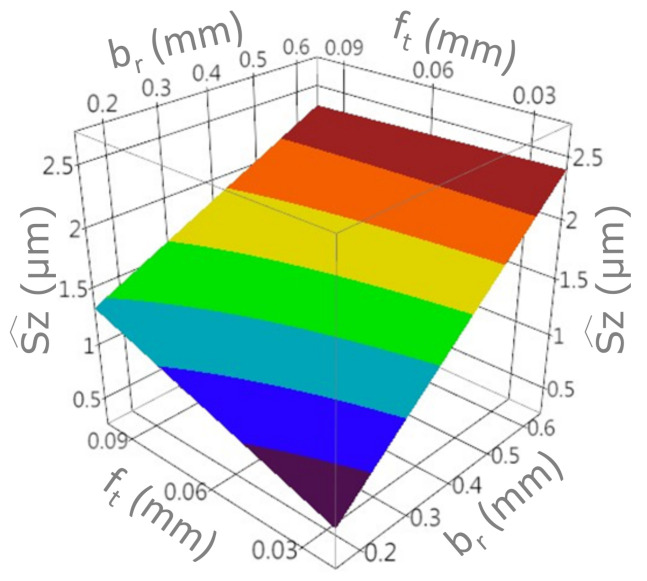
The response surface for the model with the $\hat{\mathrm{Sz}}$ output variable.

**Figure 14:**
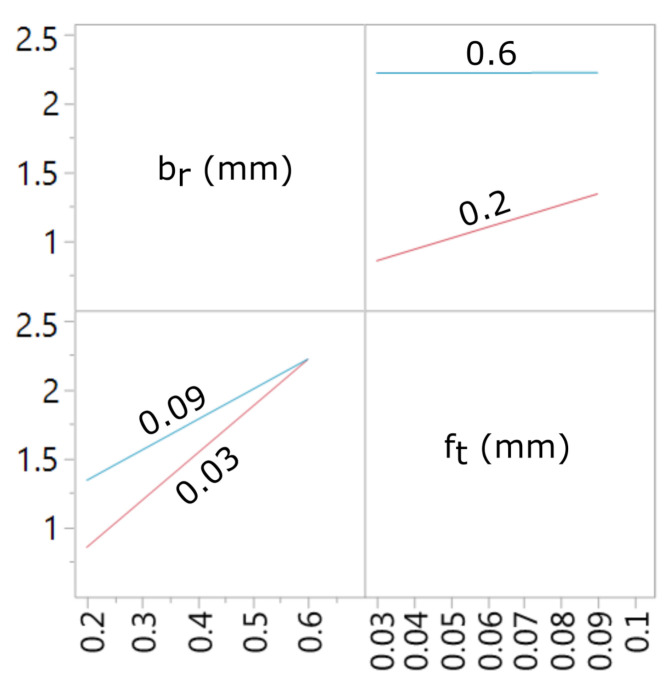
Interaction plot for a model with the $\hat{\mathrm{Sz}}$ output field.

A graphic presentation of the developed models and interaction charts are shown in the Figure 11, Figure 12, Figure 13 and Figure 14. It can be noticed that in the examined state space, the values of the analyzed parameters of the milled surface are positively correlated with the value of the feed and the cutting width. In other words, increasing the cutting width $b_{r}$ and the feed rate $f_{t}$ results in an increase in the tested parameters of the surface topography elevation features. The influence of the feed rate on the values of Sa and Sz is greater for small values of $b_{r}$. Likewise, the effect of cut width is greater with lower $f_{t}$ values. In the conducted research, the cutting width had the greatest impact on the values of the Sa and Sz surface texture parameters.

Equation coefficients for raw and standardized data and the results of the *t* test for the coefficients of the regression equation for the parameter. The conversion of data into standardized values consists in their normalization, i.e., their conversion, as a result of which their average value is 0 and the standard deviation is 1.

Empirical model for Sa and non-standardized independent variables represents the following formula:

(14) $$Sa=0.008+0.661\cdot b_{r}+0.809\cdot f_{t}-3.014\cdot \left(b_{r}-0.393\right)\cdot \left(f_{t}-0.058\right)$$

Empirical model for Sa and standardized independent variables represents the following formula:

(15) $$Sa=0.979\cdot b_{r}+0.174\cdot f_{t}-0.100\cdot \left(b_{r}-0.393\right)\cdot \left(f_{t}-0.058\right)$$

Empirical model for $\hat{\mathrm{Sz}}$ and non-standardized independent variables represents the following formula:

(16) $$\hat{Sz}=0.269+2.845\cdot b_{r}+4.215\cdot f_{t}-20.116\cdot \left(b_{r}-0.393\right)\cdot \left(f_{t}-0.058\right)$$

Empirical model for $\hat{\mathrm{Sz}}$ and standardized independent variables represents the following formula:

(17) $$\hat{Sz}=0.959\cdot b_{r}+0.206\cdot f_{t}-0.152\cdot \left(b_{r}-0.393\right)\cdot \left(f_{t}-0.058\right)$$

The mean value of the fit coefficient $R^{2}$ of the performed cross-validation was 0.94 for models with the output variable Sa and 0.93 for models with the $\hat{\mathrm{Sz}}$ output variable. Relatively large values of the fit coefficient $R^{2}$ suggest that the final models derived from all data, i.e., 30 trials, allow for good value prediction for new data, i.e., they perform well on unseen data.

**Figure 15:**
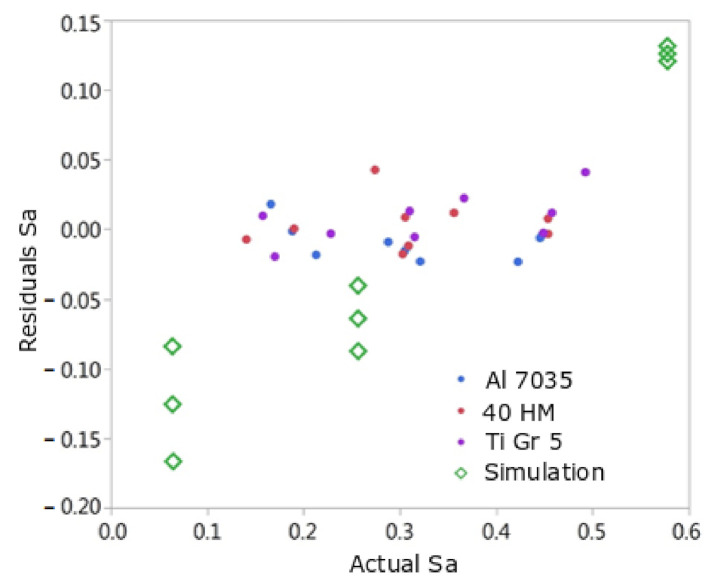
Graph of residuals depending on the real value of Sa for the tests used to build the model (Aluminum, Steel, Titanium) and the surface simulation tests after milling.

**Figure 16:**
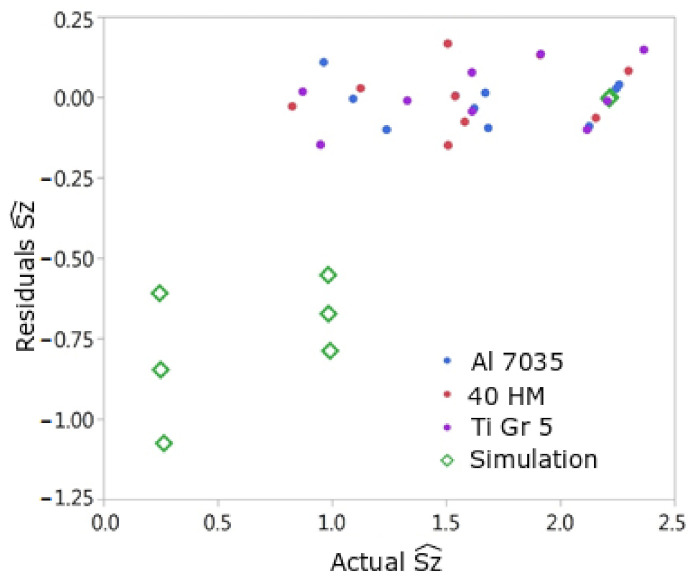
Graph of residuals depending on the real value of $\hat{\mathrm{Sz}}$ for the tests used to build the model (Aluminum, Steel, Titanium) and the surface simulation tests after milling.

Figure 15 and Figure 16 show the dependence of the model residuals on the real value (measured on samples after milling or from simulation). The residuals obtained for all performed trials of the surface simulation after milling largely differ from the empirical data, except for the three trials with the biggest value of $\hat{\mathrm{Sz}}$ parameter. The model-to-data coefficient computed for the simulated surfaces was 0.72 for the model for Sa and 0.38 for the model for the $\hat{\mathrm{Sz}}$ parameter. The results of the simulation tests, especially with regard to $\hat{\mathrm{Sz}}$, were not adequately confirmed by the results of the experimental tests. This also means that the theoretical kinematic model should be extended with additional factors that actually occur and have not been included in the simulation model so far.

In the theoretical model, even very small values of br and $f_{t}$ lead to specific modifications of the shape of treated surface in relation to untreated surface. On the other hand, in actual machining, in the case of low br and $f_{t}$ values, there are vibrations and elastic-plastic deformations. They also have the feature of periodicity resulting from variable, to some extent, process forces. Thus, the real surface after treatment is characterized by greater unevenness in relation to the theoretical model. Therefore, the empirical model transfers this feature of greater surface unevenness in relation to the theoretical model. This, in turn, results in the fact that the residual values of the measurement results in relation to the empirical model are clearly smaller than the residual values calculated from the simulation model in relation to the empirical model.

Underestimation of the values of Sa, $\hat{Sz}$ for small values of Sa, $\hat{Sz}$ by the theoretical-numerical model can, therefore, be justified by the fact of the occurrence of variable process forces and vibrations in the actual cutting process, which have not been included in the theoretical model developed so far. In turn, the overestimation of the Sa value at high Sa values by the theoretical-numerical model may probably result from the blurring of the boundaries between adjacent cutting marks.

## 4. Discussion and Conclusions

Due to the spatial nature of the studied parameters, the correlation between both important parameters, i.e., the feed $f_{t}$ and the cutting width $b_{r}$, and the observed parameters characterizing the selected high-altitude features of the surface topography Sa and $\hat{\mathrm{Sz}}$ is correct, i.e., consistent with the results of experimental studies. At small values of the cutting width $b_{r}$, the unevenness resulting from the overlapping of the machining paths is so small that the unevenness resulting from the feed movement of the cutting edge is also clearly noticeable. The increase in the profile height of the treated surface caused by the increase in the cutting width $b_{r}$ is many times greater than the value of this height resulting from the feed rate adopted in the machining process during experiments. As a result, for large values of the cutting width $b_{r}$ from the range considered in the research, i.e., for $b_{r}$ = 0.6 mm it practically eliminates the influence of the feed $f_{t}$ on observed parameters of surface texture. Moreover, note that there is no significant influence of the type of the materials used for experiments (40HM steel, Al7035 aluminum alloy and Ti Gr 5 titanium alloy) on the surface roughness. It should be emphasized, however, that in each case the cutting speed was selected individually, in accordance with the recommendations of the tool manufacturer, which served the correct course of machining.

The conducted simulation and experimental studies allow the following conclusions to be drawn.

- In the context of research on the altitude features of the surface milled topography using the lens-shaped end-mill, with the cutting width $b_{r}$ and the feed as input parameters, the choice of the surface texture Sa and $\hat{\mathrm{Sz}}$ parameters is justified from the point of view of interpretation the meaning of these parameters and the results of experimental research. The first of them, Sa is an average value and represents the arithmetical mean height of the scale-limited surface. The second, the reduced maximum height, takes into account the maximum deviations from the mean area via the sum of the parameters related to the bearing area curve $\hat{Sz}=\mathrm{Spk}+\mathrm{Sk}+\mathrm{Svk}$. In this sum, information on core roughness depth Sk, height of reduced peak height Spk and reduced depth of pits Svk is included, while the influence of information on unusual deviations from the mean surface is suppressed. The values of the adjusted fit coefficient $R_{adj}^{2}$ in the developed mathematical models turned out to be significantly large, i.e., 0.96 and 0.97. In the case of the empirical model, the parameters $b_{r}$ and $f_{t}$ and their interaction had a significant impact on the Sa and $\hat{Sz}$ parameters. The type of material and its interaction with the $b_{r}$ and $f_{t}$ parameters turned out to be statistically insignificant. Parameters $b_{r}$ and $f_{t}$ have a directly proportional and linear effect on Sa and $\hat{Sz}$. The influence of the feed rate on the values of Sa and $\hat{Sz}$ is greater for small values of $b_{r}$. Likewise, the effect of cut width is greater with lower $f_{t}$ values. With $b_{r}$ = 0.6 mm, the influence of the feed $f_{t}$ is very small. The cutting width $b_{r}$ has the greatest influence on Sa and $\hat{Sz}$, then $f_{t}$ and the interaction of these parameters has the least influence. The Stdβ standardized coefficient at the $b_{r}$ parameter is above 0.95. In contrast, Stdβ with the $f_{t}$ parameter and interactions are in the range from 0.1 to 0.2.
- In the simulation model it was possible to correctly describe basic geometry of the lens-shaped end-mill and compute the trajectories of the cutting edge movement, taking into account the radial runout, which was the basis for determining the milled surface texture, resulting from the machining kinematics. However, the adjustment of the simulation models to the experimental results is influenced by numerous disturbances occurring during the real machining process in the studied range of machining parameters. Therefore, it can be concluded that the kinematic model alone is insufficient to forecast topography and must be developed with additional factors disturbing the machining process, e.g., deformations of tool, depending on loads and time of application. Based on the simulation model, it can be concluded that the cutting width $b_{r}$ has the greatest impact on the Sa and $\hat{Sz}$ parameters. With the increase of the $b_{r}$ parameter, the Sa and $\hat{Sz}$ parameters also increased. On the other hand, the feed $f_{t}$, which had the values of 0.03, 0.06, 0.09 mm and the radial runout 0.001, 0.005, 0.01 mm, did not have a significant effect on the final value of Sa and $\hat{Sz}$. For cutting widths of 0.2, 0.4 and 0.6 mm, the Sa parameter on average had the values of 0.06, 0.26 and 0.58 μm, respectively. On the other hand, the parameter $\hat{Sz}$ had the mean values of 0.25, 0.98 and 2.22 μm, respectively.

The empirical model with a very good fit to the results of experimental measurements allows to forecast selected GSS characteristics with high accuracy after machining with a lens-shape end-mill, i.e., Sa and $\hat{Sz}$. These are altitude parameters, where Sa is the averaging parameter and $\hat{Sz}$ is the parameter approximating the maximum height. Both parameters inform about the intensity of the uplift of unevenness, and therefore they have a practical importance. Large values of Sa and $\hat{Sz}$ may lead to a greater coefficient of friction of such surfaces and their greater wear during their initial operation. Thus, it is possible to predict the operating conditions of the cooperation of objects characterized by such surfaces, e.g., the required pressures in order to obtain the appropriate friction force. Increasing the parameters Sa and $\hat{Sz}$ also negatively affects the flow resistance of the medium in the vicinity of this type of surface, e.g., in the channels of flow machines.

The developed theoretical-kinematic model is the first of the known to the public kinematic models of machining with a lens-shaped end-mill, and thus it is a novelty in this field. As shown by the comparison of the results of simulation and experimental studies, the theoretical-kinematic model is not yet perfect; however, the authors tried to explain in the modified version of the article the likely reasons for the imperfection of this model, and therefore the desired directions of its research related to its development.

The developed empirical models with very high fit coefficients reflect the results of experimental research very well. Therefore, they can be used to predict the values of selected characteristics of the geometric structure of the surface (GSS) after machining workpieces made of the materials considered in the paper with a lens-shape end-mill. This means their usefulness both from the cognitive and application points of view.

Taking into account the factors that disturb the machining process, additional to those discussed in the article, requires the development of the measuring station and the expansion of the mathematical model of the kinematics of the active edges of the tool. This will be the subject of further research by the authors.

## Figures and Tables

**Table 1:** Technological parameters used in the research.

Workpiece	Cutting	Feed per	Cutting	Cutting	Lead
Material	Speed $v_{c}$ (m/min)	Tooth $f_{t}$ (mm)	Width $b_{r}$(mm)	Depth $a_{p}$(mm)	Angle α (${}^{\circ }$)
40HM	240				
Al7035	600	0.03; 0.06; 0.09	0.2; 0.4; 0.6	0.2	4
Ti Gr 5	170				

**Table 2:** Input variables used in the research.

Variable	Value Levels
Workpiece material	40HM, Al7035, Ti Gr 5
Feed per tooth $f_{t}$ (mm)	0.03; 0.06; 0.09
Cutting width $b_{r}$ (mm)	0.2; 0,4; 0.6

**Table 3:** Equation coefficients for raw data β and standardized data Stdβ as well as the results of the *t* test for the coefficients of the regression equation for the Sa parameter.

Term	β	Stdβ	Prob>|t|
Intercept	0.008135	0	0.5595
$b_{r}$	0.660802	0.978694	<0.0001
$f_{t}$	0.809058	0.173777	<0.0001
$\left(b_{r}-0.39286\right)\cdot \left(f_{t}-0.05786\right)$	−3.014089	−0.10024	0.0058

**Table 4:** Equation coefficients for raw data β and standardized data Stdβ as well as the results of the *t* test for the coefficients of the regression equation for the $\hat{\mathrm{Sz}}$ parameter.

Term	β	Stdβ	Prob>|t|
Intercept	0.268937	0	0.0009
$b_{r}$	2.844696	0.958661	<0.0001
$f_{t}$	4.214934	0.205995	<0.0001
$\left(b_{r}-0.39286\right)\cdot \left(f_{t}-0.05786\right)$	−20.11606	−0.15223	0.0006

## Data Availability

The data presented in this study are available on request from the corresponding author.
